# Direction Matters: A Crossover Study on Motor Adaptations to Movement‐Evoked Pain Induced in the Lumbar Region

**DOI:** 10.1002/ejp.70142

**Published:** 2025-10-07

**Authors:** Valter Devecchi, Deborah Falla, Hélio V. Cabral, Jacques Abboud, Paul Hodges, Alessio Gallina

**Affiliations:** ^1^ School of Sport, Exercise and Rehabilitation Sciences, College of Life and Environmental Science University of Birmingham Birmingham UK; ^2^ Centre of Precision Rehabilitation for Spinal Pain (CPR Spine), School of Sport, Exercise and Rehabilitation Sciences, College of Life and Environmental Sciences University of Birmingham Birmingham UK; ^3^ Department of Clinical and Experimental Sciences Università degli Studi di Brescia Brescia Italy; ^4^ Département des Sciences de l'Activité Physique Université du Québec à Trois‐Rivières Trois‐Rivières Canada; ^5^ School of Health and Rehabilitation Sciences The University of Queensland Brisbane Queensland Australia

**Keywords:** experimental pain, kinematics, low back pain, motor adaptations, movement‐evoked pain

## Abstract

**Background:**

People with chronic low back pain (LBP) often experience pain evoked by movement (movement‐evoked pain [MEP]). Although pain changes how people move, it remains unclear whether motor adaptations to LBP are specific to the pain‐provocative movement. This crossover experimental study aimed to understand whether pain modulated by movement in different directions induces distinct motor adaptations, and if these adaptations are consistent with a purposeful strategy to minimise pain.

**Methods:**

Thirty healthy adults performed a repetitive box lifting task in two experimental sessions. Experimental pain was induced in the lumbosacral region using nociceptive electrical stimulation, with intensity modulated proportionally to either lumbar flexion or extension. Within‐subject changes in kinematics and centre of pressure were assessed both during and post‐pain.

**Results:**

During both sessions and over time, participants reduced their lumbar movement in the pain‐provocative direction (*p* < 0.01), but not in the non‐pain‐provoking direction (*p* > 0.078). The reduction in lumbar flexion was strongly associated with perceived pain intensity (*p* < 0.001) and persisted beyond pain resolution (*p* < 0.001). Pain during lumbar flexion also induced other acute motor adaptations, including reduced elbow flexion (*p* = 0.027) and an anterior shift of the centre of pressure (*p* < 0.001).

**Conclusions:**

This study revealed that the direction of the pain‐provocative movement is a determinant factor in motor adaptations to pain, with clinical implications in developing personalised, movement‐based interventions for LBP. Further, motor adaptations were not simply a generic acute response to pain but evolved to minimise pain, supporting the proposal that MEP is a motivational stimulus for adaptive behaviour driven by learning.

**Significance Statement:**

This study shows that motor adaptations to MEP are specific to the direction of pain‐provocative movement, evolve over time and represent a purposeful strategy to reduce pain. These findings highlight the reciprocal interactions between pain and movement, supporting the rationale for assessing motor strategies in people with movement‐evoked LBP.

## 1 Introduction

Approximately 70% of people with musculoskeletal disorders experience an increase of pain when they move, a phenomenon referred to as movement‐evoked pain (MEP) (Damsgard et al. 2010). Current theories suggest that motor adaptations to pain represent a purposeful strategy to protect the painful region from further pain and injury (Hodges and Tucker 2011). Despite the reciprocal interaction between pain and movement, research has mainly focused on how tonic pain influences movement, leaving motor adaptations to MEP largely underexplored (Corbett et al. 2019). This is particularly relevant for low back pain (LBP) given that certain movements can either exacerbate or alleviate symptoms by changing spinal loading (O'Sullivan 2005).

MEP is commonly reported by people with chronic LBP and leads to reduced motor function and disability (Knox et al. 2023). Although movement and exercise can provoke pain, they are also recommended by clinical guidelines as first‐line treatment for LBP (Foster et al. 2018). There is debate regarding whether any one approach to exercise is better than another (Grooten et al. 2022). It has been proposed that exercise that changes the way that people move is likely to be most effective for those with MEP (Hodges 2019), and recent trials supported the efficacy of this personalised approach, which uses movement‐based classification to inform treatment (Van Dillen et al. 2021). Fundamental to the hypothesis that personalised treatment is more effective than one‐size‐fits‐all approaches is that motor adaptations differ between people with LBP (Dankaerts et al. 2009; Hemming et al. 2018). For instance, pain that increases with lumbar flexion or lumbar extension may result in different changes in motor strategies because motor adaptations are thought to be a purposeful strategy to avoid pain. This association between MEP and direction‐specific motor adaptation can be tested directly in humans using experimental pain models.

In a recent systematic review, we showed that pain induced in the lumbar region results in task‐dependent changes of muscle activity and reduced lumbar range of motion (Devecchi et al. 2023). However, most of the included studies used tonic pain models, which consist of a sustained application of a nociceptive stimulus over an extended period of time and are not consistently modulated by movement. Therefore, tonic pain models cannot determine whether motor adaptation is a purposeful adaptation that is specific to the movement direction. This perspective contrasts with the clinical presentation of MEP in LBP, where movement can increase or decrease pain (Butera et al. 2016). Experimental models that use electrical stimulation enable modulation of pain intensity in real time in a manner that depends on a person's motor strategies (Cabral et al. 2023; Gallina et al. 2021). By inducing pain that increases when moving in a specific direction and reduces if the movement pattern is changed, this paradigm could test whether motor adaptation to MEP in the lumbar region is a purposeful strategy to reduce the noxious input.

This study aimed to investigate whether pain modulated by movement in different directions induces different motor adaptations, and whether these motor adaptations are a purposeful strategy to reduce pain. We hypothesised that motor adaptation would be specific to the pain‐provocative movement direction, and that such adaptations are effective at reducing pain. We also hypothesised that people would adapt upper and lower limb kinematics during experimental LBP, but that these motor adaptations would resolve over time because they were unrelated to the painful stimulation.

## 2 Methods

### 2.1 Study Design

In this crossover study, participants attended two experimental sessions separated by at least 3 days. In each session, participants performed 15 sets of a standardised box lifting task while experiencing pain experimentally induced in their lumbosacral region using noxious electrical stimulation; pain was associated with lumbar flexion in one session (Pain Flexion session), or lumbar extension in the other (Pain Extension session). To control for potential order effects, the order of sessions was randomised among participants (random.org), and the team member who enrolled participants was blind to the random allocation sequence. Joint angles of the lower body, upper body, trunk and the displacement of the centre of pressure (CoP) were the main outcomes of interest.

This study was approved by the Research Ethics Committee at the University of Birmingham, United Kingdom (ERN_19‐1018). Before the experimental procedures, all participants provided written informed consent and completed a pre‐test health screening to confirm their fitness for exercise without any contraindications. All experiments were conducted at the School of Sport, Exercise and Rehabilitation Sciences (University of Birmingham). This study is reported following the CONSORT guidelines for crossover studies (Dwan et al. 2019). We have discussed the relevance, protocol and preliminary findings of this study with members of the public and patients with chronic pain.

### 2.2 Participants

Thirty healthy volunteers (14 female, age: 23 ± 3 years; height: 172.9 ± 8.8 cm; mass: 69.3 ± 11.0 kg) were recruited from the staff and student population at the University of Birmingham, UK. The sample size was determined using G*Power, based on data from a prior study that evaluated changes in lumbar range of motion in healthy participants both before and after inducing pain in the low back region (Henchoz et al. 2013). A total of 24 participants were required to achieve an effect size (Cohen's *d*) of 0.60, a significance level (*α*) of 0.05 and a power (1 − *β*) of 0.80. To account for potential dropouts between sessions or incomplete datasets, we recruited 30 participants.

Participants were included in the study if they were aged between 18 and 50 years old. This age range was chosen to minimise potential age‐related confounders. Participants were required to have no history of lower back pain that required treatment or affected their function. Participants were excluded if they presented with contraindications to exercise (collected via a health screening questionnaire), neck, upper, or lower limb pain within the last year, a history of major spinal pathologies (i.e., infection, cancer, inflammatory disorders, fracture), prior spinal surgery, current pregnancy, or contraindication to electrical stimulation (e.g., presence of implanted medical devices or metal around the back, pelvis, or hip joints). Participants with major pathologies (neurological, neuromuscular, etc.) or those taking antidepressant drugs were also excluded.

### 2.3 Noxious Electrical Stimulation

Pain was elicited in the lumbosacral region by electrical stimulation using two surface electrodes (TE0N1S3545, SpesMedica, Genoa, Italy) placed on the sacrum at the level of S2, with a 2 cm interelectrode distance (border to border) and aligned with the midline of the spine. This location was chosen to prevent muscle twitching, and the electrodes were downsised from 35 × 45 mm to a 20‐mm diameter to further minimise current dispersion across the skin. A constant current stimulator (Digitimer DS5 Isolated Bipolar Constant Current Stimulator, Welwyn Garden City, Hertfordshire, UK) was used to deliver sinusoidal waveforms at 4 Hz. The stimulator was controlled through a custom‐written Simulink model (version 2021b, MathWorks), generating an analog signal (i.e., sinusoidal waveforms) at 2000 samples per second using a PCI‐6229 board with a 16‐bit resolution. The stimulation parameters were chosen to minimise pain habituation (Gallina et al. 2021).

The stimulation intensity was determined before the start of the lifting task by an ascending stimulation protocol in steps of 0.5 mA. During this protocol, each painful electrical stimulus was delivered for 2 s, followed by a rest period of approximately 5 s. Participants verbally rated their pain intensity using a numeric rating scale (NRS) ranging from 0 (no pain) to 10 (worst pain imaginable). The minimum and maximum stimulation intensity were identified as the stimulation amplitudes that induced a pain intensity of 1/10 and 5/10, respectively. The maximum pain intensity was similar to previous studies investigating movement‐evoked LBP in a clinical population (Rabey et al. 2017). To control for potential habituation, the stimulation intensity necessary to induce a pain of 5/10 was reassessed after every 3 sets and adjusted if necessary.

### 2.4 Lifting Task and Pain Modulation

After completing the ascending stimulation protocol, participants stood on a force plate (Kistler, 9286AA) facing a shelf, with their bare feet positioned at a standardised distance of 125% of their foot length from the shelf. The width of the stance was freely chosen by participants, and this was then recorded and maintained across all sets and sessions. Participants were asked to move a box (size: 39 × 28.5 × 16 cm, mass ~1 kg) between two shelves positioned at eye and knee level. The mass of the box was low and equal across all participants to assess the effects of pain on neuromuscular control while reducing the potential influence of confounders such as fatigue. The task mainly required movements in the sagittal plane, and no kinematic restrictions were imposed on the task. Participants were asked to continuously move the box between shelves to the pace of a metronome set at 24 beats per minute, with each beat signalling when to place the box on the lower or upper shelf. This ensured 2.5 s for both lifting and lowering of the box. A metronome was used to prevent the use of a compensatory strategy in which participants might speed up the painful phase of the movement, which is counterintuitive compared to what is typically observed for people with clinical LBP, where painful movements are commonly performed more slowly (Errabity et al. 2023). In this way, the focus was mainly on the range of movement and its redistribution across body regions. A period of familiarisation was provided before starting the assessment. In both sessions, participants completed 15 sets of 10 lifting cycles each, always starting and ending at the lower shelf. Two minutes of rest were provided between sets. Participants were aware that nociceptive electrical stimulation would not be delivered during the first two sets; in sets 3–15, they were told that they may receive the nociceptive electrical stimulation of constant or variable intensity, so they were naïve to how the stimulation was modulated. The first two sets represented the baseline condition (Base), and the recorded range of motion was used as a reference for the modulation of the stimulation in the following sets.

**FIGURE 1:**
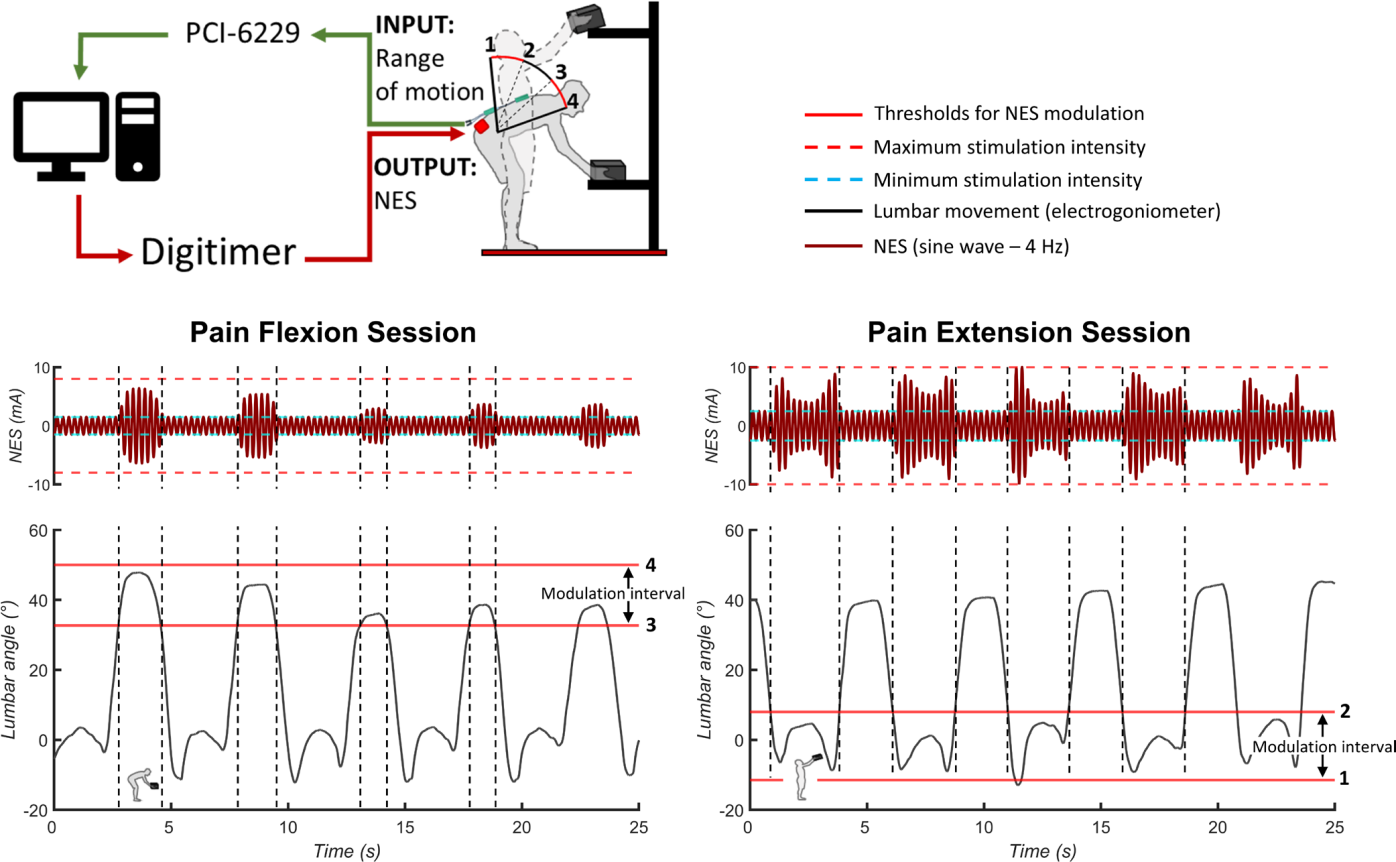
Experimental set up showing the closed‐loop associating lumbar movement (recorded by an electrogoniometer) to the delivered noxious electrical stimulation. Lumbar range of motion was collected, digitally converted by a PCI‐6229 board, and processed in real‐time using Simulink (MATLAB). During the baseline condition, the total range of lumbar movement was recorded and then used to linearly scale the delivered stimulation between its maximum and minimum stimulation intensity (dashed lines). The total range of lumbar motion (from 1 to 4) collected during baseline was evenly divided into three equal parts. In the Pain Extension session, pain modulation occurred when the lumbar movement was within the 1–2 interval. Instead, in the Pain Flexion session the pain modulation occurred when the lumbar movement was within the 3–4 interval. If the range of lumbar motion exceeded the one recorded during baseline, the stimulation intensity was limited to the maximum threshold (accordingly with the session type). NES, nociceptive electrical stimulation.

To adjust the nociceptive electrical stimulation (output) in near real‐time and proportional to the amount of lumbar movement performed (input), a closed‐loop system was implemented (Figure 1). The lumbar movement was collected by an electrogoniometer connected to a single‐channel amplifier (Forza, OT Bioelettronica, Italy) with a gain of 100 V/V and digitised with a 16‐bit converter (PCI‐6229 board, National Instruments, USA). Once digitally converted, the input signal was processed in a custom‐written Simulink model to scale the amplitude of the sine wave sent to the electrical stimulator and delivered to the participant (nociceptive electrical stimulation, output). To scale the amplitude of the sine wave, the total range of lumbar motion collected with the electrogoniometer during Base was divided into three equal intervals (flexion, neutral and extension). During Pain Flexion, for instance, the stimulation was set at the minimal pain intensity when the lumbar angle was in the neutral and extension interval, and the stimulation intensity increased linearly with the lumbar angle when the lumbar angle was within the flexion interval. If the participant reached or exceeded the maximal flexion angle recorded when performing the task without painful stimulation, the nociceptive electrical stimulation delivered was equal to the stimulation intensity that induced a pain of 5/10. After each set, participants rated their level of fatigue using the 0–10 modified Borg scale, and they rated the average intensity of pain experienced during the painful phase of the task, without providing information about when the stimulation was provided (NRS, 0–10).

### 2.5 Equipment for Movement Analysis

During the lifting task, participants were equipped with Inertial Measurement Units (Noraxon USA Inc., Scottsdale, Arizona, USA) to assess lower limb, upper limb and trunk kinematics. The IMUs were placed in accordance with manufacturer guidelines and secured with double‐sided tape. IMUs of the Noraxon have shown good reliability and concurrent validity when compared to the gold standard during both lower limb and trunk movements, especially in the sagittal plane. Specifically, joint angles showed clinically acceptable reliability (differences lower than 5*°*) and root mean square differences ranged from 1.4° to 2.6° during uniplanar movement (Berner et al. 2020; Cottam et al. 2022). A flexible electrogoniometer (M180B, Biometrics Ltd., Gwent, UK) was placed and secured using double‐sided tape on the upper and lower part of the lumbar spine (approximately between T12/L1 and L5/S1) to measure its movement in the sagittal plane; this signal was used to modulate the noxious electrical stimulation delivered by means of a pair of electrodes placed on the sacrum. A triaxial accelerometer (Noraxon USA Inc., Scottsdale, Arizona, USA) was secured in the centre of the box to identify the start and end of lifting cycles. The displacement of the CoP was the main outcome extracted from the force plate (Kistler, 9286AA, Switzerland) with signals sampled at 200 Hz and digitised with a 16‐bit converter (PCI‐6229 board, National Instruments, USA). The CoP was computed from the force and torque signals using a custom‐written Simulink model and visually inspected online.

### 2.6 Data Processing

Joint angles and box acceleration signals were sampled at 100 Hz and acquired using the myoRESEARCH software (version 3.14) and exported in MATLAB (version 2022b, MathWorks, USA) for offline processing. The signal representing the vertical acceleration of the box was smoothed with a Butterworth lowpass filter at 30 Hz (6th order). The obtained signal was differentiated to extract the jerk, and its peaks were automatically identified and visually inspected to define the start and end of each cycle. The first half of the first cycle (lifting phase) and the second half of the last cycle (lowering phase) were excluded from the analysis so that nine cycles were considered for each set (starting and ending from the upper shelf). Data from the force plate were sampled at 200 Hz and digitised using a PCI‐6229 board with 16‐bit resolution controlled by a custom‐written Simulink model for the extraction of the CoP. Once digitised, the signal from the electrogoniometer was filtered with a Butterworth 1st order lowpass filter with a cut‐off frequency of 10 Hz.

**FIGURE 2:**
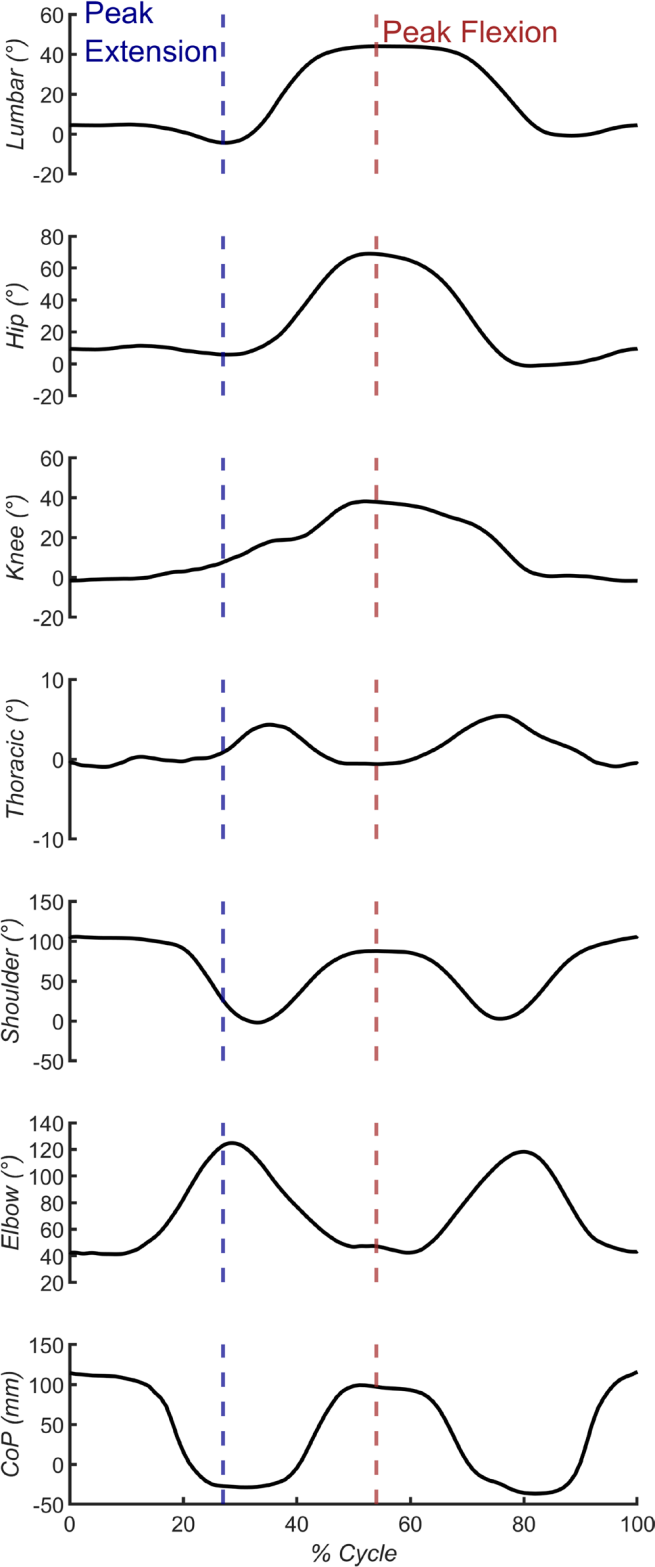
Raw data from a representative participant during the Base condition. All joint angles and the centre of pressure (CoP) along the anteroposterior axis are presented. Positive values indicate flexion movement. For the CoP, positive values indicate an anterior shift. The blue and red dashed lines indicate peak lumbar extension and flexion.

The waveform of joint angles and CoP was filtered with a Butterworth lowpass filter (4th order, 10 Hz) and divided into cycles based on the accelerometer signal. All smoothed waveforms were interpolated to create 101 samples representing 0%–100% of a lifting cycle. Within each cycle, the instants representing the peak of lumbar flexion and extension were identified and used to extract the position at the other joints at the same instants. This enabled the assessment of the motor strategy adopted by participants in relation to MEP. The peak in lumbar flexion corresponded to the instant when participants put the box on the lower shelf. The peak in lumbar extension corresponded to the phase of the cycle when participants had to move the box between their upper body and the top shelf. During each cycle, there were two peaks of lumbar extension, and the highest one was used for statistical analyses. An overview of the raw signal during Base is presented in Figure 2 from one representative participant.

The first two sets represented the Base as no pain was induced and the range of motion was used as a reference. Sets 3 and 4 represented the early adaptation condition (Early) to investigate the acute neuromuscular response to MEP, and sets 12 and 13 were the late adaptation condition (Late). Sets 14 and 15 were the post‐pain (Post) condition as the noxious electrical stimulation was not delivered.

Joint angles and CoP during both the peak in lumbar flexion and lumbar extension were extracted and averaged across cycles within the same condition (i.e., Base, Early, Late, Post) and session (i.e., Pain Flexion or Pain Extension). Similarly, perceived pain was averaged between sets 3–4 (i.e., Early) and 12–13 (i.e., Late). Borg ratings were averaged across sets within the same condition and session.

### 2.7 Statistical Analysis

All analyses were conducted in IBM SPSS Statistics (version 29.0). Based on the data normality (Shapiro–Wilk test), parametric or non‐parametric analyses were considered for inferential statistics. Different data transformations (i.e., logarithmic, square root, reverse) were applied to joint angles and CoP data based on their distribution inspected by means of QQ‐plots and histogram plots. The Shapiro–Wilk test was conducted to confirm that data were normally distributed after transformation. Greenhouse–Geisser corrections were applied to control for violations of sphericity in repeated measures. Data are reported as mean and standard deviation or median and quartiles depending on their distribution. All *p*‐values are presented after Bonferroni correction because three comparisons were assessed for each variable of interest (i.e., Early, Late and Post were compared to Base).

To evaluate the between‐session reliability of the stimulation intensity to induce a pain of 1/10 and 5/10, we used the intraclass correlation coefficient (ICC) calculated using the two‐way mixed effects model and absolute agreement for average measures. We assessed whether there was a systematic bias between sessions by comparing the maximum stimulation intensity (paired *t*‐test or Wilcoxon signed‐rank test). We used Pearson correlations to assess the agreement between lumbar flexion‐extension angles estimated with inertial sensor and electrogoniometer. This analysis was performed on the main variable of interest (change between Base–Early and Base–Late), separately for peak flexion and peak extension.

Perceived pain during Early was compared between sessions using paired t‐test or Wilcoxon signed‐rank test. The same approach was used to test within‐session changes in perceived pain between Early and Late. Perceived fatigue during each condition was compared between sessions using paired t‐test or Wilcoxon signed‐rank test. Finally, the Friedman test was used to test for differences in perceived fatigue between conditions within each session. When significant, this was followed by a Wilcoxon signed‐rank test with Bonferroni correction (three comparisons) comparing Base with the other conditions.

Joint angles and CoP during Base were compared between sessions with paired *t*‐tests or Wilcoxon signed‐rank tests. Two‐way repeated measures analyses of variance (ANOVA) were used to assess the main and interaction effects of session (Pain Flexion and Pain Extension) and condition (Base, Early, Late, Post) on joint angles and CoP. Post hoc analysis was performed as follows. When an interaction effect was present, pairwise comparisons were used to identify changes from Base within each session, for example, if in Pain Flexion the data during Early, Late, or Post differed from Base. Within‐subjects simple contrasts were used to test if the changes from Base differed between sessions, for example, if in Early the change from Base of the peak in lumbar flexion was larger during Pain Flexion compared to Pain Extension. When the main effect of conditions was significant without significant interaction with session, pairwise comparisons were applied to the main effects.

To evaluate if the adaptations in the lumbar region represent a purposeful strategy to reduce pain, we assessed if the perceived pain intensity during Late was associated with the change in lumbar kinematics between Base and Late, separately for the two sessions. The correlation was conducted using Pearson rho or Spearman rank correlation depending on data distribution, presence of outliers and linearity of the relationship.

## 3 Results

### 3.1 Stimulation Intensity, Perceived Pain and Fatigue

The stimulation intensity required to induce a pain intensity of 5/10 did not differ between Pain Extension (median [1st quartile, 3rd quartile]: 8.5 [5.6, 12.5] mA) and Pain Flexion (8.0 [5.1, 12.0] mA) sessions (difference: 0.25 [−1.0, 0.5] mA; Wilcoxon signed‐rank test, *N* = 30, *z* = 1.48, *p* = 0.140). ICC values [95% confidence interval] to evaluate between‐session reliability were 0.85 [0.68, 0.93] for the stimulation intensity needed to induce a pain intensity of 5/10, and 0.60 [0.19, 0.81] for the stimulation intensity needed to induce a pain of 1/10.

Correlation analyses revealed that the change from Base to Early showed a Pearson correlation of *r* = 0.81 for peak flexion and *r* = 0.83 for peak extension between Noraxon and electrogoniometer measures. For the change from Base to Late, the Pearson correlations were *r* = 0.83 for peak flexion and *r* = 0.66 for peak extension.

Perceived pain during Early did not differ between Pain Extension (3.22 ± 1.01) and Pain Flexion (3.18 ± 1.13) sessions (*t* = −0.12, *p* = 0.90). During Pain Flexion, a reduction of perceived pain intensity from Early (3.18 ± 1.14) and Late (2.68 ± 1.33) conditions narrowly missed significance (difference: −0.5 ± 1.44, *t* = 1.90, df = 29, *p* = 0.068). Changes were characterised by large inter‐individual variability; out of 30 participants, 12 reported a reduction of perceived pain of at least 1 out of 10, 14 reported minimal changes (smaller than ±1 out of 10), and 4 reported higher pain during Late compared to Early. No difference in perceived pain was observed during Pain Extension between Early (3.22 ± 1.01) and Late (3.13 ± 1.54) conditions (difference: 0.09 ± 1.39, *t* = 0.36, df = 29, *p* = 0.72). Also for this condition, large variability in the responses was observed among participants with 9 reporting a reduction of perceived pain of at least 1 out of 10, 12 reported minimal changes (smaller than ±1 out of 10), and 9 reported higher pain during Late compared to Early. During all conditions, perceived fatigue did not differ between sessions (*p* > 0.40). Additionally, within both sessions, perceived fatigue differed between conditions (*p* < 0.001), but the median increase in perceived fatigue between Base and Post was minimal (Pain Extension: 0 [0–1.38], *p* < 0.001, and Pain Flexion: 0.63 [0–1], *p* < 0.001).

### 3.2 Effects of Movement‐Evoked Pain on Joint Angles and Centre of Pressure

**FIGURE 3:**
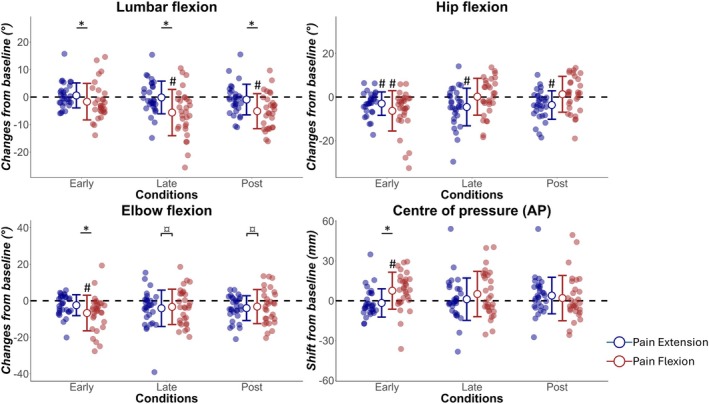
Changes from Base for multiple joint angles and centre of pressure during the peak in lumbar flexion. Data are presented for all participants. *Interaction effect (session × condition) with significant planned contrasts between sessions (*p* < 0.05 after Bonferroni correction). #Pairwise comparisons revealed a significative difference from Base (*p* < 0.05 after Bonferroni correction). ¤Presence of a main effect of condition, with pairwise comparisons showing a significant difference from Base when sessions are pooled together (*p* < 0.05 after Bonferroni correction).

**FIGURE 4:**
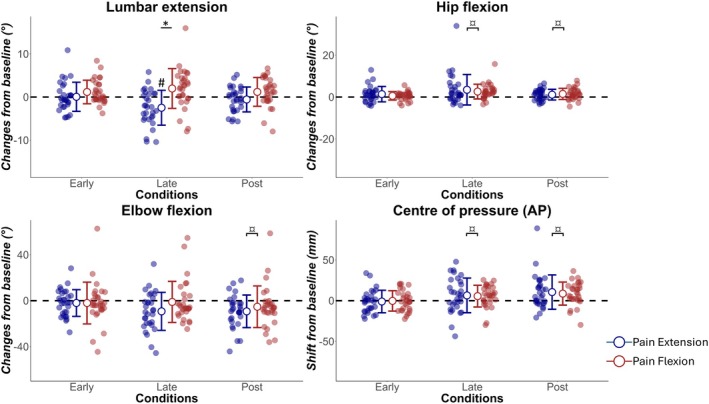
Changes from Base for multiple joint angles and centre of pressure during the peak in lumbar extension. Data are presented for all participants. *Interaction effect (session × condition) with significant planned contrasts between sessions (*p* < 0.05 after Bonferroni correction). #Pairwise comparisons revealed a significate difference from Base (*p* < 0.05 after Bonferroni correction). ¤Presence of a main effect of condition, with pairwise comparisons showing a significant difference from Base when sessions are pooled (*p* < 0.05 after Bonferroni correction).

**FIGURE 5:**
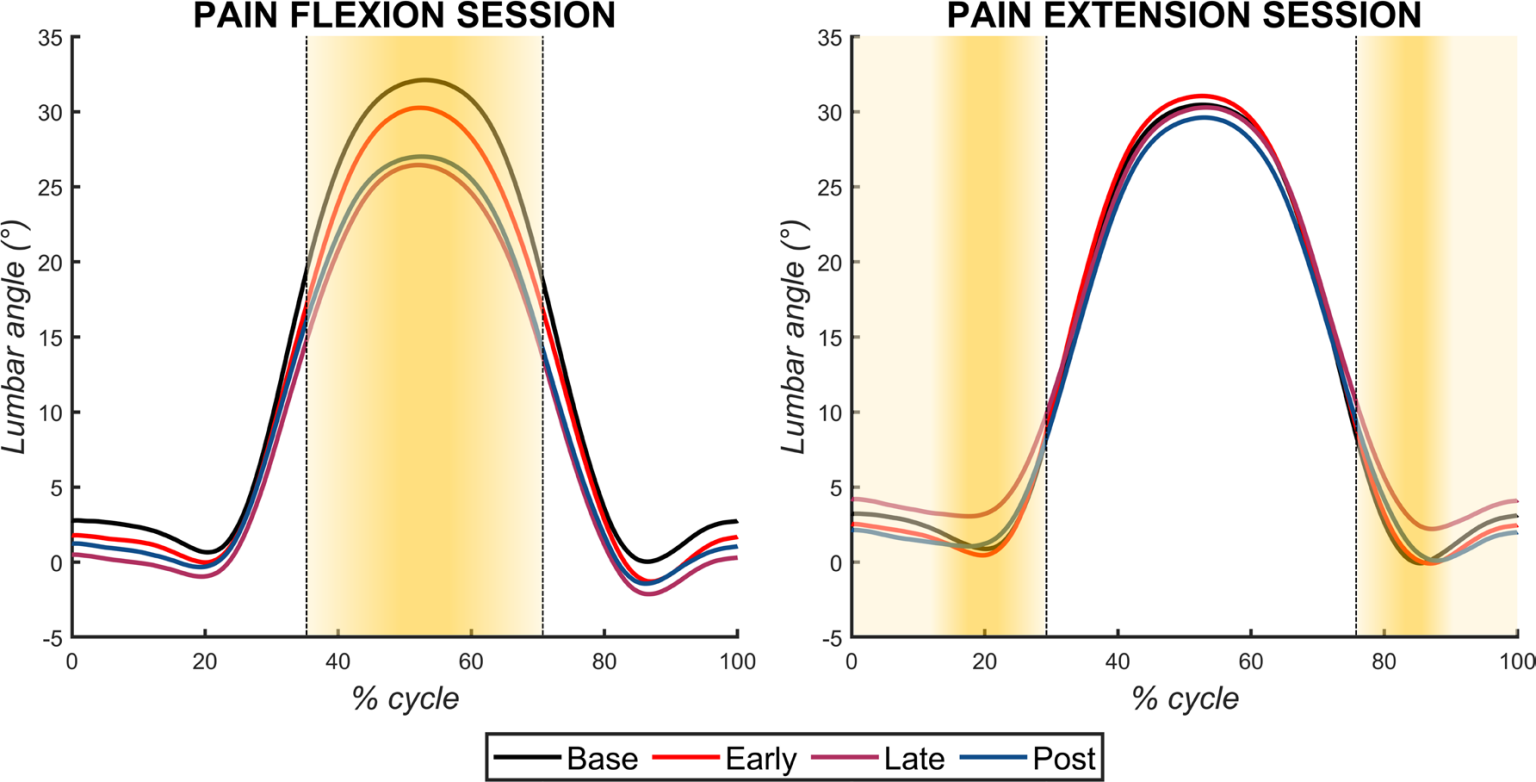
Average waveforms of the lumbar angle across conditions during both the Pain Flexion and Pain Extension sessions. The area in yellow represents the pain modulation interval. Compared to Base, a reduction in the lumbar flexion peak is present during the Pain Flexion session across all conditions, especially during Late and Post‐Pain. Although small, in the Pain Extension session there is a reduction of the extension peak in the Late condition compared to Base.

Data from all participants were included in the analyses. No outcome of interest differed at Base between Pain Flexion and Pain Extension sessions during the peak in lumbar flexion (*p* > 0.11) or the peak in lumbar extension (*p* > 0.34). Outcomes of interest with different patterns of changes between the two sessions are illustrated in Figure 3 (peak lumbar flexion) and Figure 4 (peak lumbar extension). Average waveforms of the lumbar angle during both sessions and for different conditions are presented in Figure 5. All values are reported in the Supporting Information S1. Below, the analyses on the data extracted at peak lumbar flexion and at peak lumbar extension are presented separately. As a reminder, peak lumbar flexion refers to the flexion component of the task, and it was the pain provocative phase of movement during the Pain Flexion session. In the opposite direction, peak lumbar extension refers to the extension component of the task, and it was the pain provocative phase of movement during the Pain Extension session. The direction of changes is summarised in Table 1.

**TABLE 1:** Changes of kinematic features and centre of pressure (CoP) compared to baseline.

	Outcome of interest	Early		Late		Post	
		Pain flexion	Pain extension	Pain flexion	Pain extension	Pain flexion	Pain extension
Peak in lumbar flexion	Lumbar flexion	↓	—	↓	—	↓	—
	Hip flexion	↓	↓	—	↓	—	↓
	Knee flexion	—	—	—	—	—	—
	Thoracic flexion	—	—	—	—	—	—
	Shoulder flexion	—	—	—	—	—	—
	Elbow flexion	↓	—	↓	↓	↓	↓
	CoP (*anterior shift*)	↑	—	—	—	—	—
Peak in lumbar extension	Lumbar extension	—	—	—	↓	—	—
	Hip flexion	—	—	↑	↑	↑	↑
	Knee flexion	—	—	—	—	↓	↓
	Thoracic flexion	—	—	—	—	—	—
	Shoulder flexion	—	—	—	—	—	—
	Elbow flexion	—	—	—	—	↓	↓
	CoP (*anterior shift*)	—	—	↑	↑	↑	↑

*Note:* Red cells indicate different adaptations between sessions, as determined by planned contrasts.

### 3.3 Peak in Lumbar Flexion

Two‐way repeated measures ANOVA identified an interaction effect of session and condition on lumbar angle (*F*(3, 87): 6.74, *p* = 0.004). Pairwise comparisons in Pain Flexion revealed a reduction of lumbar flexion compared to Base during Late (−5.63 ± 8.41, *p* < 0.001) and Post (−5.13 ± 6.36, *p* < 0.001), but not during Early (−1.67 ± 6.63, *p* = 0.53). No changes compared to Base were observed during Pain Extension (*p* = 1). Planned contrasts support a larger reduction of peak lumbar flexion when pain was modulated in flexion than when pain was modulated in extension (Early: *F*(1, 29) = 8.23, *p* = 0.008; Late: *F*(1, 29) = 10.42, *p* = 0.009; Post: *F*(1, 29) = 7.73, *p* = 0.027). Despite the significant difference with Base and between sessions, the reduction of lumbar flexion during Late was characterised by large inter‐individual variability. Specifically, of the 30 participants tested, 7 showed minimal changes (within ±2°), 19 a reduction, and 4 an increase of lumbar flexion compared to Base. Of those who reduced lumbar flexion, only one participant returned to a range of motion similar to Base during Post.

Hip angle data were assessed after logarithmic transformation. Two‐way repeated measures ANOVA identified an interaction effect of session and condition on hip angle (*F*(3, 87): 6.78, *p* < 0.001). Pairwise comparisons revealed that during Early, participants performed the task with reduced hip flexion during both Pain Flexion (−6.31 ± 9.26, *p* = 0.009) and Pain Extension (−2.99 ± 5.36, *p* = 0.015). During Pain Extension, the reduction of hip flexion was also present during Late (−4.56 ± 8.61, *p* = 0.033) and Post (−3.65 ± 6.52, *p* = 0.018) whereas in Pain Flexion, no changes were observed (*p* = 1). The reduction of hip flexion between Base and other conditions did not differ between sessions (planned contrasts: *p* > 0.14).

Elbow angle data were assessed after logarithmic transformation. Two‐way repeated measures ANOVA identified an interaction effect of session and condition on the elbow angle (*F*(3, 87) = 5.26, *p* = 0.004). During Pain Flexion, pairwise comparisons identified a lower elbow flexion in Early compared to Base (−6.74 ± 9.74, *p* = 0.006). The reduction of elbow flexion was larger during Pain Flexion than Pain Extension during Early (planned contrast: *F*(1, 29) = 7.83, *p* = 0.027), but did not differ in Late (*F*(1, 29) = 0.002, *p* = 1) or Post (*F*(1, 29) = 0.81, *p* = 1). Two‐way repeated measures ANOVA identified a main effect of condition on elbow angle (*F*(3, 87) = 5.63, *p* = 0.005). Pairwise comparisons revealed a difference from Base for both Late (*F*(1, 29) = 10.23, *p* = 0.009) and Post (*F*(1, 29) = 14.33, *p* < 0.001).

Data of the CoP were assessed after square root transformation. Two‐way repeated measures ANOVA identified an interaction effect of session and condition for the CoP (*F*(3, 87): 7.51, *p* < 0.001). Pairwise comparisons identified an anterior shift of the CoP during Early compared to Base during Pain Flexion (7.56 ± 13.93, *p* = 0.006). No changes were found for other conditions (*p* > 0.77) or during Pain Extension (*p* > 0.67). The anterior shift of the CoP was larger during Pain Flexion than Pain Extension but only during Early (planned contrast: *F*(1, 29) = 14.71, *p* < 0.001).

Two‐way repeated measures ANOVA identified no main effect of condition or interaction effect for the knee (assessed after square root transformation, *p* > 0.236), thoracic (*p* > 0.146) and shoulder (*p* > 0.098) angles.

### 3.4 Peak in Lumbar Extension

Two‐way repeated measures ANOVA identified an interaction effect of session and condition on lumbar angle (*F*(3, 87): 7.84, *p* = 0.001). Pairwise comparisons in Pain Extension revealed a reduction of lumbar extension compared to Base during Late (−2.48 ± 4.04, *p* = 0.006) but not during Early (0.06 ± 3.37, *p* = 1) or Post (−0.56 ± 2.88, *p* = 0.87). No changes compared to Base were observed during Pain Flexion (*p* > 0.078). A planned contrast comparing Base with Late showed a larger reduction of lumbar extension during Pain Extension than Pain Flexion (*F*(1, 29) = 13.38, *p* = 0.003). Similarly to the peak in lumbar flexion, large inter‐individual variability was observed in the changes between Late and Base. Changes during Late were minimal (within ±2°) in 12 participants, 16 showed a reduction and two an increase of lumbar extension. Of the 16 participants who reduced lumbar extension, six returned to a range of motion similar to or larger than Base, and 10 maintained the reduced lumbar extension during Post.

Some changes were also observed in joints other than the low back. Hip angle data were assessed after square root transformation. Two‐way repeated measures ANOVA identified a main effect of condition on hip angle (*F*(3, 87): 8.40, *p* < 0.001). Compared to Base, participants performed the task with more hip flexion during both Late (*F*(1, 29) = 13.28, *p* = 0.003) and Post (*F*(1, 29) = 13.4, *p* < 0.001). Knee angle data were assessed after square root transformation. A main effect of condition was found (*F*(3, 87) = 4.83, *p* = 0.011). Pairwise comparisons revealed that during Post participants performed the task with a reduction of knee flexion (*F*(1, 29) = 15.41, *p* < 0.001). Similarly, two‐way repeated measures ANOVA identified a main effect of condition on elbow angle (*F*(3, 87) = 5.75, *p* = 0.002). Pairwise comparisons showed a reduction of elbow flexion compared to Base during Post (*F*(1, 29) = 12.65, *p* = 0.003). Although a main effect of condition on thoracic angle was found (*F*(3, 87): 6.41, *p* = 0.002), pairwise comparisons after Bonferroni corrections did not reveal any difference between Base and other conditions (*p* > 0.078). Two‐way repeated measures ANOVA identified no main effect of condition or interaction effect on shoulder angle (*p* > 0.475).

Two‐way repeated measures ANOVA identified a main effect of condition on the CoP (*F*(3, 87): 12.51, *p* < 0.001). Compared to Base, a larger anterior shift of the CoP was observed during both Late (*F*(1, 29) = 7.02, *p* = 0.039) and Post (*F*(1, 29) = 18.27, *p* < 0.001).

### 3.5 Correlation Between Perceived Pain and Kinematics

**FIGURE 6:**
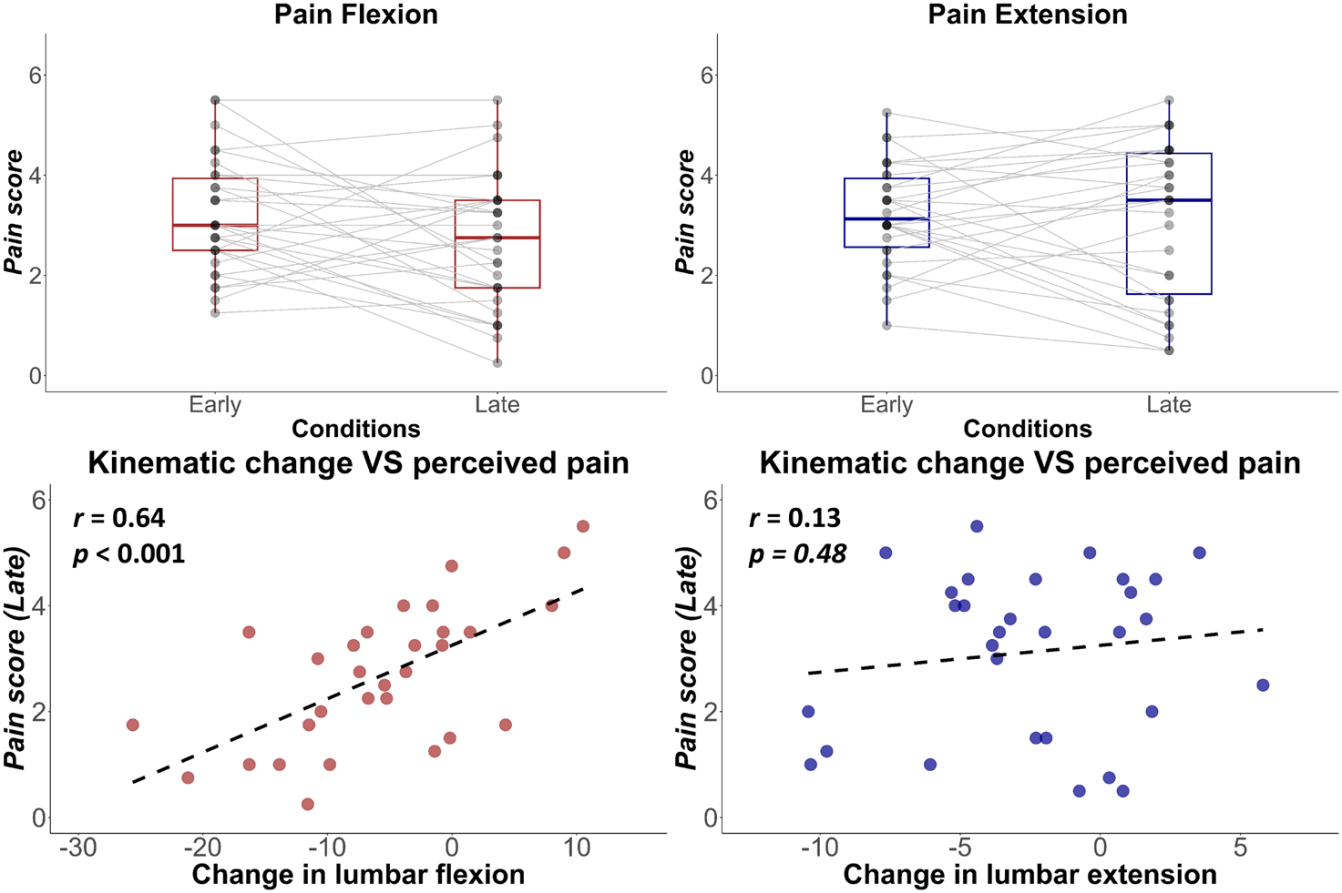
Top: Perceived pain and its changes between Early and Late conditions. Bottom: association between changes in lumbar kinematics (Base—Late) and perceived pain during Late. All data are presented for the Pain Extension (blue) and Pain Flexion (red) sessions.

To evaluate if the adaptations in the lumbar region are a purposeful strategy to reduce pain, and to investigate whether variability in lumbar kinematic adaptations can explain the variability in pain intensity in Late, the association between pain intensity and lumbar kinematic changes was assessed (Figure 6). During Pain Flexion, a strong correlation was identified between the change from Base to Late of the lumbar flexion peak and the amount of perceived pain reported during the Late condition (*t* = 4.37, df = 28, *p* < 0.001, *r* = 0.64). During Pain Extension, there was no significant correlation between changes in lumbar extension peak and perceived pain (*t* = 0.71, df = 28, *p* = 0.48, *r* = 0.13).

## 4 Discussion

When exposed to experimentally induced MEP, participants reduced their range of lumbar movement in the pain‐provoking direction. The association between a larger reduction of lumbar flexion and lower perceived pain supports the notion that the adaptations represent a purposeful strategy to reduce pain. Our findings show that experimental pain in the same location and of similar intensity induces different motor adaptations depending on which movements modulate pain intensity. These data suggest that the provocation of pain by movement is a determinant of motor adaptations to pain.

### 4.1 Motor Adaptations to Experimentally Induced MEP Evolve Over Time and Become Specific to the Pain‐Provocative Movement

When the experimentally induced pain increased with lumbar flexion, participants first adopted a multi‐segment strategy, but over time the adaptations became limited to the pain‐provoking lumbar movement only. This suggests that motor adaptations to MEP change as information regarding the relationship between pain and movement is acquired. The specificity of adaptation to the pain‐provoking movement was also observed over time when experimental pain was induced during lumbar extension. Acute, non‐specific adaptations induced by pain associated with lumbar flexion suggest that participants relied more on their arms to lift the box and reduced the distance between the centre of mass and the box, possibly to reduce the moments acting on the spine (Jäger and Luttmann 1989; Schipplein et al. 1995). Although a recent systematic review did not find clear evidence for remote adaptations in response to tonic lumbar pain (Devecchi et al. 2023), lower limb pain resulted in remote adaptations during tasks that involved multiple degrees of freedom (Brøchner Nielsen et al. 2017; Hug et al. 2014; Simonsen et al. 2019). This could explain why we did not observe remote adaptations when pain was delivered during lumbar extension, where biomechanical constraints limit the available solutions to complete the task. Our data suggests that, when multiple biomechanical solutions are available, and until a strategy to reduce pain is identified, the central nervous system prioritises a redistribution of load along the kinematic chain to unload and protect the painful area.

Participants reduced the range of the painful movement over time, suggesting that they needed multiple repetitions to learn the association between movement and modulation of pain. Changes in motor adaptation over time in response to painful electrical stimulation have been described in several studies, and include progressively more delayed deep trunk muscles activation (Moseley and Hodges 2005), a progressive restoration of the baseline heel contact duration during gait (Bertrand‐Charette et al. 2021), and a progressive unloading of the painful leg during quiet stance (Gallina et al. 2021). Such learning processes can be described by the ‘pain perception – action cycle’ where sensorimotor integration, informed by memory, enables the development of an internal prediction model that aims to anticipate and avoid the pain provocative movement (Chen and Wang 2023). Thus, the observed adaptation can also be interpreted within a classical conditioning framework, where the experience of pain (unconditioned stimulus) paired repeatedly with a specific movement (conditioned stimulus) triggered a defensive response – in this case, a direction‐specific movement behaviour (i.e., conditioned response) (Meulders 2020). Overall, our findings from a healthy population suggest that acute pain is a motivational stimulus for adaptive behaviour driven by learning.

The reduction of lumbar movement with pain aligns with the common observation of reduced range of lumbar flexion in subgroups of patients with clinical LBP (Errabity et al. 2023) and in response to tonic experimental pain (Devecchi et al. 2023). Our study adds that motor adaptation observed in the short term is specific to the phase of the movement in which pain is experienced and that only the range of motion in the painful direction is restricted. The specificity of this motor adaptation aligns with neurophysiological evidence that, during motor preparation, corticospinal excitability of a muscle decreases when acting as an agonist to perform a painful movement, but it increases when acting as an antagonist to the painful movement (Neige et al. 2018). Compared to the multi‐segment adaptation observed in the acute phase, a more selective adaptation has the advantage to minimise nociceptive input while also limiting the metabolic costs associated with generalised motor adaptation (van Dieën et al. 2017). Reduction of lumbar ROM was consistent across participants, likely because it was the only solution to reduce the painful stimulus. However, as we did not measure muscle activity, it remains unclear whether this reduction in lumbar ROM was due to a decreased activation of the agonist muscles, an increased activation of the antagonist muscles, or to the general stiffening response often observed in people with clinical and experimentally induced LBP (Devecchi et al. 2023; Van Dieën et al. 2003). In contrast to adaptations in the lumbar region, a large inter‐individual variability was observed for the other joints, likely because of the existence of different biomechanical solutions to compensate for the reduced lumbar motion. Our data suggest that participants exposed to MEP selectively restrict the movement that induces pain, at least when experiencing an acute noxious stimulus.

In contrast to most studies using tonic pain models, we observed motor adaptations that outlasted pain duration during lumbar flexion. Such findings suggest that although provocation of pain is a motivator to adapt, removal of the nociceptive stimulus does not motivate a return to the original movement strategy, as has been suggested previously (Hodges and Tucker 2011). Pain‐related fear conditioning has been proposed as a possible mechanism for the development of avoidant behaviour that persists even when the nociceptive stimulus is removed, suggesting that pain‐related fear might be key in promoting and sustaining the observed motor adaptation (Meulders 2020). It is plausible that motor adaptation might persist until pain‐related fear is addressed and new information is acquired confirming that the once painful movement is now pain‐free (Seymour et al. 2023). Although our findings are limited to the short term, long‐term maintenance of altered movement strategies despite symptom remission (Devecchi et al. 2021) could lead to deconditioning, suboptimal loading and restriction of sensory information from the lumbar region (Hodges and Smeets 2015), factors which could contribute to pain persistence (Hodges and Tucker 2011). Future studies may use a similar methodology to assess whether movement restriction and avoidance generalise beyond the originally painful task to other tasks that share similar biomechanical, perceptual, or contextual factors in the long term (Vandael et al. 2023).

### 4.2 Are Motor Adaptations to MEP a Purposeful Strategy to Reduce Pain?

Our results partially support the hypothesis that changes in movement behaviour are a purposeful adaptation to reduce pain. When pain was associated with lumbar flexion, the reduction in lumbar flexion was strongly correlated with lower perceived pain intensity. However, this was not observed when pain was associated with lumbar extension. This might have several explanations: (i) the reduction of flexion movement was twice that observed during extension; (ii) the flexion movement could be accomplished with a greater variation of motor solutions that were available in extension, which allowed participants to redistribute the effort and movement across multiple joints; (iii) participants received one long painful stimulation per cycle during flexion and two short stimulations during extension. Our findings provide new evidence that motor adaptation is consistent with a purposeful strategy to limit pain. Previous research has attempted to address this question by inducing tonic pain in different body locations. That approach produced conflicting evidence with studies supporting (Gallina et al. 2018) or refuting (Falla et al. 2009; Hug et al. 2013) the notion that motor adaptation is specific for pain location. A possible reason for these findings is that tonic pain, although specific to a region, cannot be reduced by changing the movement strategy and thus removes the critical element of exposure to a less painful option. Here we provide evidence that, for an identical pain location, participants over time learned to limit the painful movement, and this resulted in low pain (at least for lumbar flexion) for those who adapted. This supports the notion that the goal of motor adaptation is to limit pain, at least in the short term (Hodges and Tucker 2011), and highlights the role of painful movements as a determinant of motor adaptation to pain.

### 4.3 Parallels With Clinical Low Back Pain and Potential Implications

Although the experience of experimental pain in a laboratory setting differs from the experience of individuals with clinical LBP in several respects (e.g., psychosocial elements of clinical pain), we observed a range of motor adaptations that share some similarities with the movement patterns observed in clinical populations. Based on the number of participants who reduced their range of motion over time, and whether this motor adaptation was resolved in the absence of painful stimulation, our data show that: (i) some individuals failed to identify a motor strategy that reduces their pain; (ii) some individuals identified a motor strategy that reduces their pain, but the motor adaptation persisted beyond pain resolution; and (iii) some individuals identified a motor strategy to reduce their pain, and gradually returned to the original motor pattern as their pain subsided. The first scenario presents similarities to patients who continue to perform the movement in a way that contributes to their experience of pain (provocative and maladaptive motor response), potentially leading to task avoidance. The second scenario, instead, involved the use of a protective motor strategy that restricts the painful movement even after pain has resolved, with potential long‐term negative consequences. It is notable that we observed such variation in adaptations despite a highly standardised, repeatable painful stimulus. Although we did not assess psychological factors such as pain‐related fear, variation in these factors between individuals could account for some of the between‐participant variation in motor adaptations and their evolution over time. Evidence from both clinical LBP and pain‐free populations suggests that pain‐related fear can influence lumbar movement in lifting tasks (Christe et al. 2021; Knechtle et al. 2021). Overall, motor adaptations to experimentally induced pain observed in this study mirror some of the strategies commonly reported for individuals with clinical LBP. Our findings support the rationale for personalised interventions that address patient‐specific dysfunctional pain‐provocative movements or aim to restore movement in a manner that is less provocative (Van Dillen et al. 2021; Wernli et al. 2020, 2022).

### 4.4 Methodological Considerations

The recruitment of a sample consisting primarily of young adults without LBP limits the interpretation and transferability of findings to clinical populations. It is without question that clinical LBP is more complex than the discrete short‐lived pain experience possible in a laboratory setting. Many factors such as comorbid pain in multiple body regions, heightened nervous system sensitivity, and psychological distress are likely to contribute to motor adaptations in a manner that would underpin large heterogeneity. Further, in contrast to our young adult participants, individuals who are less familiar with movement and exercise might find it more challenging to identify an effective less‐painful strategy. Our experimental pain model served as a tool to assess a specific mechanism for motor adaptation, and the observation of high variation in the absence of these confounding factors highlights that heterogeneity is observed in a tightly constrained exposure. Future studies in clinical populations are necessary to confirm whether pain directionality is a determinant of motor adaptations to MEP, to identify factors that might limit the discovery of effective compensatory motor strategies, and to investigate whether people with clinical LBP show greater pain‐related fear or a higher propensity to generalise movement avoidance across different tasks.

Although noxious electrical stimulation lacks spatial specificity due to the activation of both nociceptive and non‐nociceptive neurons, it represents a unique method to assess the effects of pain modulated by movements in opposite directions. Noxious electrical stimulation cannot fully reproduce the qualitative characteristics and natural feeling experienced by those with clinical LBP. For example, our experimental pain model delivered pain mainly at the end of the range of motion, and although this would replicate some clinical presentations, individuals with clinical LBP might also experience pain at the beginning of moving in extension from a flexed position, within mid‐range postures and other permutations. The moderate correlation between inertial sensors and electrogoniometer for peak extension from Base to Late might have limited the magnitude of the observed motor adaptation and also influence the correlation between reduced lumbar extension and perceived pain.

## 5 Conclusions

These findings support the importance of pain directionality in determining motor adaptations to MEP. Pain experienced in the same location and of a similar intensity induced different motor adaptations over time depending on the direction of movement that provoked the pain. Our findings provide new insights on the reciprocal interaction between pain and movement, and provide mechanistic support for future clinical investigations of people with LBP.

## Author Contributions

**Valter Devecchi, Deborah Falla, Jacques Abboud, Paul Hodges** and **Alessio Gallina:** conceptualization. **Valter Devecchi** and **Hélio V. Cabral:** investigation. **Valter Devecchi:** formal analysis. **Alessio Gallina, Deborah Falla, Jacques Abboud** and **Paul Hodges:** funding acquisition. **Valter Devecchi:** writing – original draft. **Valter Devecchi, Deborah Falla, Hélio V. Cabral, Jacques Abboud, Paul Hodges** and **Alessio Gallina:** writing – review and editing.

## Conflicts of Interest

The authors declare no conflicts of interest.

## Supporting information


**Table S1:** ejp70142‐sup‐0001‐Supinfo01.docx.
**Table S2:** ejp70142‐sup‐0001‐Supinfo01.docx.

## Data Availability

The datasets used for the analyses is available from the corresponding author upon reasonable request.
