# Supplementary material for: Direction Matters: A Crossover Study on Motor Adaptations to Movement‐Evoked Pain Induced in the Lumbar Region
Source: Eur J Pain. 2025 Oct 7;29(10):e70142. doi: 10.1002/ejp.70142 (PMC12501826; doi:10.1002/ejp.70142)
Supplement: Supplementary file 1 — Table S1: ejp70142‐sup‐0001‐Supinfo01.docx. Table S2: ejp70142‐sup‐0001‐Supinfo01.docx. [file EJP-29-0-s001.docx]

**SUPPLEMENTARY FILES**

| **Table S1.** Joint angles and center of pressure (CoP) during the peak of lumbar flexion (mean ± SD) | | | | | |
| --- | --- | --- | --- | --- | --- |
| ANGLE | SESSION | **CONDITIONS** | | | |
|  |  | BASE | EARLY | LATE | POST |
| Lumbar (°) | *Pain Flexion* | 32.5 ± 11.7 | 30.8 ± 11.9 | 26.9 ± 12.6 | 27.4 ± 11.4 |
|  | *Pain Extension* | 30.8 ± 11.8 | 31.4 ± 12.4 | 30.7 ± 13.3 | 29.9 ± 13.2 |
| Hip (°) | *Pain Flexion* | 50.4 ± 19.8 | 44.1 ±21.9 | 50.5 ± 22.0 | 51.7 ± 21.8 |
|  | *Pain Extension* | 52.9 ± 20.0 | 49.9 ± 21.1 | 48.3 ± 18.8 | 49.3 ± 18.8 |
| Knee (°) | *Pain Flexion* | 21.3 ± 24.2 | 21.6 ± 26.6 | 24.4 ± 30.9 | 20.9 ± 26.5 |
|  | *Pain Extension* | 23.6 ± 25.9 | 22.2 ± 26.0 | 19.1 ± 21.6 | 18.5 ± 20.5 |
| Thoracic (°) | *Pain Flexion* | 12.9 ± 8.1 | 11.8 ± 11.2 | 11.9 ± 7.6 | 14.3 ± 10.5 |
|  | *Pain Extension* | 12.3 ± 8.0 | 11.5 ± 8.0 | 13.3 ± 9.6 | 12.0 ± 8.6 |
| Shoulder (°) | *Pain Flexion* | 55.2 ± 14.1 | 52.0 ± 14.0 | 53.0 ± 9.8 | 52.2 ± 11.2 |
|  | *Pain Extension* | 58.2 ± 9.2 | 59.0 ± 10.8 | 55.4 ± 13.5 | 57.0 ± 11.9 |
| Elbow (°) | *Pain Flexion* | 26.8 ± 12.5 | 20.1 ± 12.5 | 23.5 ± 14.0 | 23.7 ± 14.2 |
|  | *Pain Extension* | 29.5 ± 15.9 | 27.0 ± 15.0 | 25.3 ± 15.3 | 25.4 ± 14.8 |
| CoP (mm) | *Pain Flexion* | 50.2 ± 30.5 | 57.8 ± 31.4 | 55.3 ± 26.8 | 52.2 ± 25.3 |
|  | *Pain Extension* | 49.7 ± 33.9 | 48.0 ± 31.5 | 50.8 ± 27.2 | 53.6 ± 27.2 |

| **Table S2.** Joint angles and center of pressure (CoP) during the peak of lumbar extension (mean ± SD) | | | | | |
| --- | --- | --- | --- | --- | --- |
| Angle | Session | **CONDITIONS** | | | |
|  |  | BASE | EARLY | LATE | POST |
| Lumbar (°) | *Pain Flexion* | -2.3 ± 4.5 | -3.5 ± 5.1 | -4.3 ± 6.3 | -3.5 ± 5.7 |
|  | *Pain Extension* | -2.3 ± 5.1 | -2.3 ± 5.1 | 0.2 ± 6.2 | -1.7 ± 4.5 |
| Hip (°) | *Pain Flexion* | 1.3 ± 3.2 | 1.9 ± 3.9 | 3.9 ± 4.3 | 2.8 ± 3.4 |
|  | *Pain Extension* | 1.8 ± 3.8 | 3.2 ± 3.4 | 5.3 ± 6.9 | 2.9 ± 3.2 |
| Knee (°) | *Pain Flexion* | 4.7 ± 3.5 | 3.8 ± 3.2 | 3.3 ± 3.3 | 3.3 ± 3.4 |
|  | *Pain Extension* | 4.8 ± 3.4 | 4.9 ± 3.7 | 5.6 ± 6.0 | 3.3 ± 3.2 |
| Thoracic (°) | *Pain Flexion* | -7.6 ± 5.1 | -5.8 ± 5.5 | -7.9 ± 5.7 | -8.5 ± 5.6 |
|  | *Pain Extension* | -7.8 ± 5.7 | -7.5 ± 5.0 | -8.9 ± 4.9 | -8.1 ± 5.1 |
| Shoulder (°) | *Pain Flexion* | 52.3 ± 17.4 | 49.4 ± 15.7 | 52.6 ± 15.9 | 53.2 ± 15.8 |
|  | *Pain Extension* | 53.4 ± 12.9 | 53.1 ± 14.4 | 55.7 ± 15.4 | 58.5 ± 14.8 |
| Elbow (°) | *Pain Flexion* | 83.5 ± 17.3 | 81.5 ± 21.3 | 82.4 ± 19.2 | 78.2 ± 19.2 |
|  | *Pain Extension* | 86.6 ± 18.6 | 84.6 ± 19.9 | 77.4 ± 22.1 | 77.4 ± 20.5 |
| CoP (mm) | *Pain Flexion* | -24.3 ± 23.0 | -24.5 ± 24.5 | -18.7 ± 26.9 | -15.6 ± 27.4 |
|  | *Pain Extension* | -24.9 ± 25.8 | -25.9 ± 28.4 | -18.4 ± 28.0 | -14.3 ± 25.6 |
